# Ecology of planktonic ciliates in a changing world: Concepts, methods, and challenges

**DOI:** 10.1111/jeu.12879

**Published:** 2021-12-22

**Authors:** Thomas Weisse, David J.S. Montagnes

**Affiliations:** ^1^ Research Department for Limnology University of Innsbruck Mondsee Austria; ^2^ Department of Evolution, Ecology, and Behaviour University of Liverpool Liverpool UK

**Keywords:** climate change, food webs, gene expression, mixotrophy, numerical and functional response, plankton models, protists, rare biosphere, thermal response, trophic diversity

## Abstract

Plankton ecologists ultimately focus on forecasting, both applied and environmental outcomes. We review how appreciating planktonic ciliates has become central to these predictions. We explore the 350‐year‐old canon on planktonic ciliates and examine its steady progression, which has been punctuated by conceptual insights and technological breakthroughs. By reflecting on this process, we offer suggestions as to where future leaps are needed, with an emphasis on predicting outcomes of global warming. We conclude that in terms of climate change research: (i) climatic hotspots (e.g. polar oceans) require attention; (ii) simply adding ciliate measurements to zooplankton/phytoplankton‐based sampling programs is inappropriate; (iii) elucidating the rare biosphere's functional ecology requires culture‐independent genetic methods; (iv) evaluating genetic adaptation (microevolution) and population composition shifts is required; (v) contrasting marine and freshwaters needs attention; (vi) mixotrophy needs attention; (vii) laboratory and field studies must couple automated measurements and molecular assessment of functional gene expression; (viii) ciliate trophic diversity requires appreciation; and (ix) marrying gene expression and function, coupled with climate change scenarios is needed. In short, continued academic efforts and financial support are essential to achieve the above; these will lead to understanding how ciliates will respond to climate change, providing tools for forecasting.

For over a century, plankton ecologists have focused on forecasting, making predictions associated with applied and environmental outcomes, from fish recruitment to climate change. Here, we review how an appreciation of planktonic ciliates has become central to these predictions. Admittedly, ciliates are ecologically important in many other environments (e.g. wetlands, sewage systems, as endo‐ and ecto‐symbionts; Esteban & Fenchel, 2021; Lynn, 2008), but as 70% of our planet is covered by water, where much of the world's carbon flux occurs, planktonic systems seem an appropriate focus.

Our aim is not to overwhelm the reader by documenting the extensive body of works that has led to the appreciation for planktonic ciliates; we have, therefore, refrained—mostly—from naming the hundreds (possibly thousands) of researchers who have contributed to the field (see Table 1 for a brief summary and guide to the literature). Rather, we explore the process by which these works have recognized ciliates as key players in pelagic food webs; a steady progression, punctuated by a combination of conceptual insights and technological breakthroughs (Figure 1). By reflecting on these key events, we hope to offer suggestions as to where future leaps are needed and are likely to occur. Specifically, we identify what we see are the needed steps for ciliate research in the era of global warming, the world's most pressing ecological challenge.

**TABLE 1:** Milestones of planktonic ciliate research; a very brief guide to the literature (original articles in regular font, review articles in *italics*)

Year/period	Method(s)	Application	Conceptual progress	Orig. references/*Reviews*
1674–1850	Light microscopy	Detection and taxonomy	Ciliates as “little animals” (animalcula)	van Leeuwenhoek 1674, cited by *Dobell*, *1932* and *Corliss*, *1975*; Müller, 1779; Ehrenberg, 1838; *Corliss*, *1979*
1751	Binomial nomenclature		Taxonomy and systematics	Linnaeus, 1751
1880s	Plankton nets	First crude quantification	Def. of ‘Plankton’ (1887)	Hensen, 1887
1886	Theoretical approach	Ecological research; taxonomy	Def. of ‘Ecology’, ciliates are protists, not “little animals”	Haeckel, 1866
1900–1920	Bottle sampling, pumping, and filtration, centrifugation	Improved quantification of small cells (< 50 µm)	Classical grazer food chain	Hensen, 1887; Hensen, 1895; Kofoid, 1897; Lohmann, 1908; Lohmann, 1911; *Beers*, *1982*; *Wasmund et al*., *2008*
1935–1980	Utermöhl method (Lugol's fixation and inverted microscopy)	Accurate quantification of abundance and biomass	Ciliates are quantitatively important in aquatic food webs	Utermöhl, 1931; Utermöhl, 1958; Lund et al., 1958; *Edler & Elbrächter*, *2010*
1970–1985	Epifluorescence microscopy (Acridin orange, DAPI…)	Quantification of heterotrophic and autotrophic bacteria (picoplankton) and small nanoplankton	Microbial loop, size‐structured (microbial) food web	Hobbie et al., 1977; Porter & Feig, 1980; Pomeroy, 1974; Azam et al., 1983; *Porter et al*., *1979*; *Sherr & Sherr*, *1984*; *Kemp et al*., *1993*
1980s	Dilution technique, FLB^a^/FLC^a^/FLA^a^/FLCil^a^ methods, size‐fractionation and diffusion chambers	Rate measurements	Ciliates are important grazers of algae and bacteria and may also prey upon other ciliates	Beers & Stewart, 1971; Bloem et al., 1989; Dolan & Coats, 1991; Landry & Hassett, 1982; Müller & Weisse, 1994; Rublee & Gallegos, 1989; Sherr et al., 1987; Šimek et al., 1995; Stoecker et al., 1983; *Kemp et al*., *1993*
1970–1995	Coulter counters and Analytical flow cytometry (AFC)	Automated analyses of microbial abundance and biomass in empirical and experimental studies	Smooth planktonic biomass spectrum declining with size; improved quantification of microbial standing stocks and rates	Burkill, 1987; Cucci et al., 1985; Kenter et al., 1996; Lavin et al., 1990; Olson et al., 1985; Reckermann & Colijn, 2000; Sheldon et al., 1972; Sheldon et al., 1986; Yentsch et al., 1983; *Burkill*, *1987*; *Reckermann & Colijn*, *2000*
1985–1995	Quantitative protargol staining (QPS)	Estimates of ciliate abundance, biomass, and diversity	Improved taxonomic resolution of quantitative analyses	Montagnes & Lynn, 1987; Skibbe, 1994
1980s	Culturing of planktonic ciliates	Estimates of growth, predation, and grazing loss rates; numerical and functional responses; thermal performance; biomass conversion factors	Confirming the significance of ciliates as predators and prey; ciliates as model organisms for ecophysiological and evolutionary research	Gifford, 1993; Gifford, 1985; Montagnes, 1996; Montagnes et al., 2008; *Kemp et al*. *(* *1993* *)*; *Montagnes et al*., *2012*; *Weisse*, *2006*; *Weisse et al*., *2016*
1985–2000	Large collaborative sampling programs and international meetings	Data collection and interpretation	Ciliates as key players in microbial food webs; structural differences between the ocean and lakes	Gaedke, 1992; Garrison et al., 2000; Lessard & Murrell, 1996; Müller et al., 1991; Stoecker et al., 1994; Weisse et al., 1990
1985–2015	Methods reported above; primary productivity estimates; modeling	Food web models	Mixotrophy is widespread in ciliates and important for the ‘biological carbon pump’ in the ocean	Laval‐Peuto et al., 1985; Mitra et al., 2014; Ward & Follows, 2016; Woelfl & Geller, 2002; *Beaver & Crisman*, *1989*; *Stoecker*, *1991*; *Stoecker et al*., *2017*
1995–2015	Imaging flow cytometry, FlowCAM	Improved automated analyses of microbial abundance and biomass of large data sets; visual evidence of key players	Improved analyses of α‐diversity and β‐diversity and seasonality	Álvarez et al., 2014; Buskey & Hyatt, 2006; Jakobsen & Carstensen, 2011; Sieracki et al., 1998
2005–	Trait‐based ataxonomic approaches	Large data sets; ecophysiology; predictive models (e.g. for climate change scenarios)	Community and ecosystem ecology; assessing functional redundancy, ecosystem stability, and resilience	Gravel et al., 2016; Le Quere et al., 2005; McGill et al., 2006; Violle et al., 2017; Zhong et al., 2017; *Weisse et al*., *2016*; *Weisse*, *2017*
2010–	DNA/RNA barcodes, high‐throughput sequencing (HTS)	Estimates of ciliate diversity	Ciliate inter‐ and intraspecific diversity may be much higher than revealed by form (morphospecies)	Forster et al., 2019; Gimmler et al., 2016; Pitsch et al., 2019; Santoferrara & McManus, 2020; Stoeck et al., 2014; *Caron & Hu*, *2018*; *Caron et al*., *2012*; *Gao et al*., *2017*; *Lynn*, *2008*; *Santoferrara et al*., *2020*
2010–	(Single cell) genome sequencing, metagenomics, metatranscriptomics	Phylogeny; genetic complexity; functional diversity; symbiotic and other protist**–**protist interactions**;** response to environmental drivers	Detecting metabolic pathways; understanding the functional role in the ecosystem context, macroevolution and microevolution of ciliates	Keeling et al., 2014; Kolisko et al., 2014; Lima‐Mendez et al., 2015; Yoon et al., 2011; *Caron et al*., *2017*; *d'Alelio et al*., *2019*; *del Campo et al*., *2014*

^a^ Fluorescently labeled bacteria (FLB), cyanobacteria (FLC), algae (FLA) and ciliates (FLCil).

**FIGURE 1:**
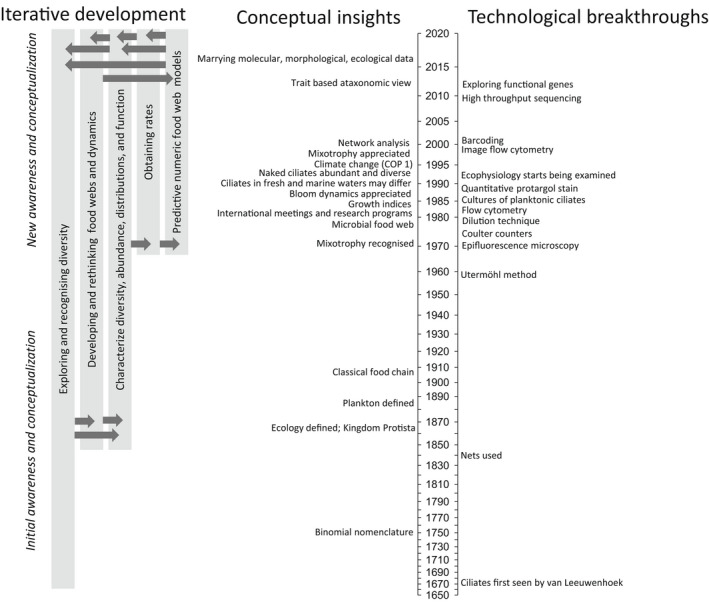
A timeline of the study of planktonic ciliates, indicating the iterative development of the field through technological breakthroughs leading to conceptual insights, and vice versa. The “Gantt chart” to the left of the figure follows the timeline on the right, indicating the temporal progression of approaches. The arrows indicate the interconnectedness of iterations, as new awareness has led to the pursuit of data and knowledge, and again vice versa; note that placement of arrows is not related to the timeline but is simply for convenience of the design. The timeline on the right—which is on a log‐scale to emphasize the extensive developments in recent years—juxtaposes technological breakthroughs and conceptual insights, and by doing so reveals temporal iterations between them. Of note is the gap between 1920s and the 1950s, reflecting the political upheaval of the time. For more details and references, see Table 1

Much of scientific research, including this journey with ciliates, follows progressive steps, from initial awareness (e.g. through natural history) toward synthesis and application. But these steps rarely progress linearly through time. Instead, as new insights arise they stimulate the development of novel techniques for exploration, while new methodologies, developed sometimes for entirely different fields, are often seized and adapted by open‐minded researchers. This intertwined interaction between insights and innovations is exemplified in the study of planktonic ciliates.

Below, we first offer a brief history prior to the 1980s of how insights and innovations led to a better understanding of planktonic ciliates (a more detailed review of this period is presented by Beers, 1982). We then provide an overview of the insights leading onwards from the 1980s and then expand on these by highlighting the main technological advances leading to them. Ultimately, reflecting on this iterative progression, we suggest where new insights and innovations are needed to address how planktonic ciliates will respond to climate change, and hence impact the ecosystem.

## INITIAL AWARENESS AND CONCEPTUALIZATION

Ciliate research began 350 years ago, with the first technological advance: Leeuwenhoek's microscope (Figure 1, see Corliss, 1975) provided awareness that free‐living ciliates are ubiquitous, occurring in water bodies ranging in volume from raindrops to the ocean. These observations, coupled with such conceptual developments as the establishment of binomial nomenclature by Linnaeus in the mid‐1700s and recognition of “ecology” and “protists” by Haeckel in the late 1800s (see Table 1), led to further appreciation of ciliates. Early works on ciliates in the 18th and 19th centuries were descriptive and taxonomic (Table 1), including remarks on lifestyle, occurrence, and distribution of ciliates (Müller, 1779, see Dolan, 2013); but they did not assess their abundance.

First attempts to quantify microorganisms in the pelagic ecosystems were made in the mid and late 19th century. The first nets were developed by J. Müller and were applied by Viktor Hensen in the Baltic Sea, using 53‐μm silk mesh (Müllergaze No.20; reviewed by Wasmund et al., 2008). This approach to quantification became popular in North America, Norway, Austria, and elsewhere (Birge & Juday, 1922 and references therein), but it soon became clear that small cells (< 50 µm) were not caught (Table 1, Kofoid, 1897). In his effort to quantify all planktonic organisms, Lohmann (1908, 1911) explored several modifications, such as pumping and filtration, bottle sampling, and centrifugation (see Wasmund et al., 2008 for an English summary). The extensive studies of Hensen, Kofoid, and Lohmann (Table 1) paved the way to quantify smaller plankton.

**FIGURE 2:**
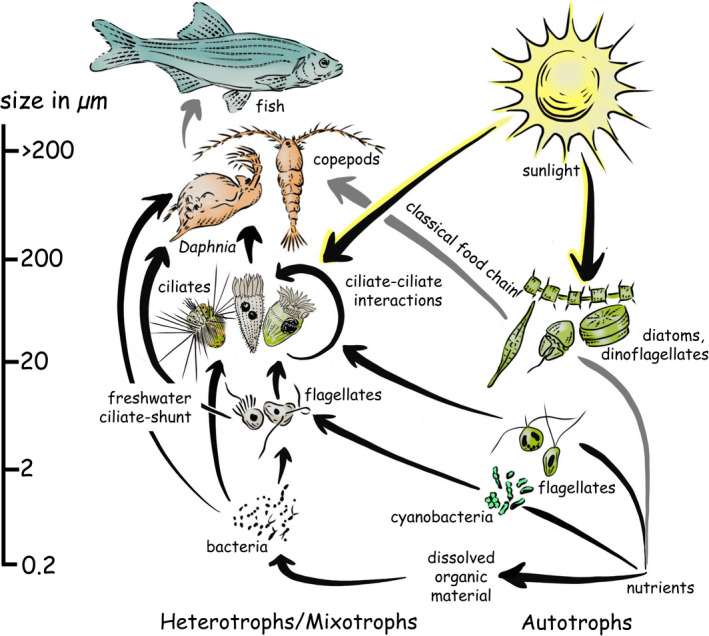
The position and role of ciliates within the planktonic food web of marine and fresh waters, liberally adapted from Esteban and Fenchel (2021). Illustrations are clearly not to scale, and the scale bar on the far left reflects organismal size. Heterotrophs and mixotrophs are presented on the left and autotrophs are presented on the right (we have for the purposes of this review ignored the fact that dinoflagellates are predominantly mixotrophic). The classical food chain—from nutrients (e.g. nitrogen, phosphorous, silica), to large phytoplankton (e.g. diatoms and dinoflagellates), to mesozooplankton (e.g. copepods and cladocera), and then to planktivorous fish—is depicted by grey arrows. The microbial food web is reflected by all arrows. All other labels should be self‐explanatory or are referred to in the text. Of particular note are: (i) the “freshwater ciliate‐shunt,” where both auto‐ and heterotrophic flagellates and bacteria are often consumed directly by cladocera, which tend to be better competitors for these prey when they are abundant, with carbon flow then bypassing (or shunting around) the ciliates, (ii) “ciliate‐ciliate interactions”, which include predation and possibly parasitism, leading to recycling within the ciliate assemblage, and (iii) “mixotrophy” by ciliates, allowing them to act as primary producers. For details, see the main text and references in Table 1

This quantification of plankton led to conceptualizing the planktonic food chain (Lohmann, 1908, 1911): primary producers (phytoplankton) dominate in the sea and lakes, and were fed upon by small herbivores (copepods or cladocerans), which in turn were preyed upon by carnivorous zooplankton, ultimately providing food for small fish. These insights led to the first solid understanding of food web dynamics, what we now call the classical grazer food chain (Figure 2, grey arrows). With some refinement (Lindeman, 1942), this model was so attractive and simple that it remained entrenched in aquatic ecology for over 50 years, restricting our wider understanding until the late 1970s (see Further awareness and conceptualization, below).

Lohmann (1911) and his contemporaries had already begun to recognize the importance of smaller taxa, including ciliates. Tintinnid ciliates, which possess a hard lorica, were sampled by nets, and some soft‐bodied ciliates were also captured (Dolan, 2013). Lohmann (1911) also saw the role of bacteria primarily as decomposers. There was even then a growing recognition of the rapid growth rates of bacteria and protozoa, but at that time bacteria and protozoa were believed to be quantitatively unimportant, relative to phytoplankton (Weisse, 2003). Two key events changed this view and led to a recognition that the classical food chain was an inadequate construct.

The first advance was a systematic investigation of the significance of ciliates in the second half of the 20th century, using the Utermöhl method (Utermöhl, 1958; Table 1). Utermöhl designed and popularized methods for quantifying phytoplankton using settling chambers as early as the late 1920s, but this practice did not become common, standardized, or strongly applied to ciliates until the 1950s (Lund et al., 1958). The Utermöhl method was then applied to count both nanoplankton (2–20 µm) and microplankton (20–200 µm), which were poorly assessed using nets and inadequately preserved with formaldehyde (a widely used fixative for phytoplankton, Sournia, 1978). Instead, in the Utermöhl method plankton samples are carefully collected in bottles, preserved with Lugol's solution, concentrated in sediment chambers, and quantified using an inverted microscope (Edler & Elbrächter, 2010; Sournia, 1978). With some modifications, this method, which revealed an unexpected abundance of ciliates, remains a mainstay of many modern sampling programs (Edler & Elbrächter, 2010; Gifford & Caron, 2000).

The second advance was due to a combination of methodological developments in the 1960s and 70s primarily associated with bacteria and small flagellates, not ciliates (epifluorescence microscopy, sampling methods, and culturing techniques, see Gifford & Caron, 2000; Kemp et al., 1993). These led to a revolutionary rethinking of the role of bacteria and nanoplankton in planktonic food webs, resulting in our current appreciation of the size‐structured microbial food web and how the classical food web is imbedded within it (Figure 2, all arrows; Azam et al., 1983; Pomeroy, 1974; Sheldon et al., 1972; Sieburth et al., 1978).

Based on the new methods, researchers were able to recognize that ciliates play important roles in this revised food web, acting as food for mesozooplankton, consumers of bacteria and algae, and nutrient remineralizers (Porter et al., 1979; Sherr & Sherr, 1984); i.e. they were neither simply “heterotrophic phytoplankton” nor tiny “zooplankton.” The next step was to build a better understanding of these diverse roles through data collection and method development, a second era of awareness and conceptualization. Below, we artificially separate the iterative processes leading to a better appreciation of planktonic ciliates by first presenting the conceptual leaps in this era; then, we indicate how these were arrived at through observations and innovations.

## NEW AWARENESS AND CONCEPTUALIZATION

The 1980s were a time for rethinking and gathering of like‐minded researchers. For instance, in 1981 (NATO/CNSR funded in Villefranche‐sur‐Mer, France) and then in 1988 (NATO funded, in Plymouth, UK), there were international meetings that brought together many of the marine and freshwater experts who studied protozoa and specifically ciliates in pelagic food webs. In the years following such meetings, several developments are of particular note (Figure 1, Table 1). We briefly introduce these below, without discussing how they arose.

One particularly important conceptual realization was that the microbial food web is similar but not identical in fresh and marine waters (Figure 2). Planktonic ciliates—which mainly consume nanoplankton (2–20 µm)—occur in both systems but play different roles primarily due to the different dominant mesozooplankton (200–2000 µm). In marine waters, calanoid copepods dominate, and these grazers are inefficient at consuming prey < 10 µm (Kiørboe, 2011; Paffenhöfer, 1998), but may consume ciliates. While in freshwaters Cladocera, primarily *Daphnia*, dominate, and they are capable of grazing nanoplankton and even some picoplankton (Gliwicz, 2003; Weisse, 2003). In both cases, *Daphnia* compete with ciliates for algal and bacterial food. Bactivorous ciliates usually require relatively high bacterial abundances (> 1–3 × 10^6^ cells ml^‐1^) and are, therefore, more important in eutrophic freshwater systems than in the oligotrophic ocean (Beaver & Crisman, 1989; Pierce & Turner, 1992; Šimek et al., 1996). Consequently, in marine waters, ciliates are a key link between nanozooplankton and mesozooplankton, while in freshwaters, there is a ciliate‐shunt (Figure 2): i.e. ciliates are competitors with the dominant mesozooplankton, and often poor ones at that, hence prey are “shunted” past ciliates. Ciliates are, thus, trophically important in most marine waters but are only so in freshwaters when top–down control (e.g. by fish) removes cladocerans or in oligotrophic systems where low algal and bacterial biomass cannot support *Daphnia* (Weisse, 2003). This dichotomy of food web structure, based on meso‐grazers has profound implications (see Jürgens, 1994; Sommer & Sommer, 2006; Weisse, 2003).

A second rethinking of the microbial food web was a recognition of the importance of mixotrophy, in both marine and freshwaters. Many planktonic ciliates are able to retain prey chloroplasts and use them to photosynthesize, to various extents (Esteban et al., 2010; Stoecker, 1991; Stoecker et al., 2017). It has become clear that mixotrophy is widespread and increases trophic transfer efficiency, vertical carbon flux, and nutrient recycling (Mitra et al., 2014; Ward & Follows, 2016). The contribution of ciliates to mixotrophic carbon sequestering in the sea may be substantial considering that 30%–40% of the marine oligotrichs are mixotrophs (Stoecker et al., 2017 and references therein). To our knowledge, such a global estimate is not available for freshwater species, but mixotrophs may reach at times 25%–50% of the total ciliate abundance in lakes (Sanders, 2011) and appear to be of comparable quantitative importance in oligotrophic freshwater environments to that in the open sea (Esteban et al., 2010; Tittel et al., 2003).

Another major final conceptual revision is not so much the recognition of a single phenomenon but rather an appreciation of the complexity of the microbial food web and the role of ciliates within it, and specifically ciliate–ciliate interactions (Figure 2). For instance, raptorial ciliates may predate on other ciliates (Dolan & Coats, 1991; Yang et al., 2021) and ciliates may be parasitized by other protists (Coats & Bachvaroff, 2013; Weisse & Sonntag, 2016). These complex interactions of symbionts, pathogens, and predators have led to a revised paradigm of the “multifarious lifestyles of microbes” (Worden et al., 2015). As we indicate further below, the wide application of culture‐independent molecular tools in the new millennium has provided insights into several of the above interactions. Commensurate with this understanding has been a shift from the traditional, taxonomic‐oriented approach toward an ataxonomic, trait‐based functional approach to understanding the ecology of ciliates (Weisse, 2017; Weisse et al., 2016). This new focus on function, merging form with genes and gene expression, represents another rethinking of the microbial food web concept (Clamp & Lynn, 2017).

## HOW DID WE GET THERE?

In parallel, and leading to, conceptualizing the structure of the microbial‐based food web (Figure 2), there were concerted efforts to determine the key players, their abundance, and their ecological functions (often referred to as ecophysiology). Collecting data was paramount. Some of the field data had been collected before the 1980s, mainly from easily accessible regions (e.g. coastal areas and temperate lakes), but efforts were needed to assess how ciliates fit within the new concept of a microbial food web at a global level.

### Characterizing biodiversity of taxa, abundance, distributions, and function based on form

Stimulated by the new concepts, there was an intensive characterization of planktonic ciliate diversity and distribution in fresh and marine waters between the 1980s and 2000s (Foissner et al., 1999; Sherr & Sherr, 1984). These efforts were in a large part the result of funding being channeled into large marine sampling programs, such as the Joint Global Ocean Flux Study (JGOFS, 1987–2003), which encouraged, at a regional scale, similar research in lakes (e.g. the collaborative research program “Cycling of Matter in Lake Constance” 1986–1997).

Using mainly the Utermöhl method, ciliate abundance and biomass estimates (determined from biovolumes, Gifford & Caron, 2000; Menden‐Deuer & Lessard, 2000) were reported from many regions of the world, supporting their quantitative significance (Gaedke, 1992; Lynn & Montagnes, 1991; Rodriguez & Mullin, 1986). The rapidly accumulating data revealed new trends that allowed us to better understand the food web: (1) ciliate abundances in the euphotic regions of both lakes and oceans are on the order of 1–10 ml^−1^, but occasional high abundances > 100 ml^−1^ suggested rapid bloom dynamics, both temporal and seasonal (Lynn & Montagnes, 1991; Montagnes, 1996); in a seminal paper, Smetacek (1981) revealed that ciliates preceded copepods in the spring bloom, revolutionizing our understanding of seasonal bloom dynamics—ciliates are now embedded in conceptual models of plankton succession (Sommer et al., 2012); (2) small ciliates (~10–50 µm) dominate in the open sea and most (oligotrophic) lakes, i.e. the prey size that is preferentially ingested by copepods and cladocerans (see previous section); (3) ciliates compete with microcrustaceans and rotifers for nano‐sized prey (McManus & Santoferrara, 2013; Weisse, 2006); (4) ciliate diversity is high and dominated by “naked” oligotrichs and choreotrichs (ranging in size from ~10 to 100 µm), with the tintinnids— previously thought to be the major ciliates—representing only 0%–5% of the marine ciliate community (McManus & Santoferrara, 2013) and contributing very little to ciliate diversity in lakes (Dolan, 2013; Foissner et al., 1999).

Determining ciliate diversity, however, was hampered by technical and conceptual issues. First, Lugol's solution, used in the Utermöhl method, although an excellent fixative, reveals only superficial features, preventing detailed taxonomic identification. The taxonomic resolution was improved and new diversity was recognized when protargol staining, a mainstay of the morphological characterization of ciliates, was made quantitative (QPS, Montagnes & Lynn, 1987). However, as QPS is labor intensive and protargol stain is no longer commercially available, this approach has dwindled in its application. Furthermore, as the Utermöhl method and QPS tend to assess samples of tens to a few hundreds of milliliters, rare species may have gone unnoticed. Likewise, “cryptic” species may not be revealed by morphological studies (Fenchel, 2005; Fenchel et al., 1997). The ubiquity of the “rare biosphere” (Caron & Countway, 2009; Dunthorn et al., 2014) has only begun to be more fully appreciated with the advent of genetic tools and their application to assessing ciliates (see Characterizing biodiversity of taxa, abundance, distributions, and function based on genes).

Over the years, there have also been concerted efforts to automate counting, with mixed results. Early on Coulter^®^ Counters, and similar electronic counters, were used to assess abundances and size distributions (Sheldon et al., 1972, 1986). With the development of flow cytometers and their application to fieldwork, electronic counters were replaced (Burkill, 1987; Reckermann & Colijn, 2000). More recently, at least for plankton in the size range of ciliates, imaging flow cytometers, most notably FlowCAM^®^, have been applied to examine ciliates (Álvarez et al., 2014; Jakobsen & Carstensen, 2011; Sieracki et al., 1998). A clear advantage of these automated techniques is the increase in sampling and hence better spatial/temporal resolution with less effort. Secondly, FlowCAM measurements can be performed with live cells, avoiding artifacts of fixation and preservation (Jakobsen & Carstensen, 2011; Zarauz & Irigoien, 2008). Thirdly, imaging flow cytometry is a superb tool for analyzing trait–environment relationships (e.g. predator‐dynamics) of planktonic protists (Pomati et al., 2013; Weisse et al., 2016). The drawback of these methods is that even with image capturing flow cytometers, the taxonomic resolution is poor, although new methods may be forthcoming to improve this (Kerr et al., 2020).

### Characterizing biodiversity of taxa, abundance, distributions, and function based on genes

PCR‐based methods used since the mid‐1980s have been modified to asses genetic variation in ciliates (summarized by Caron et al., 2012; Lynn, 2008). For instance, the mitochondrial cytochrome *c* oxidase I (*cox1*), “barcode,” gene, has been used to assess the interspecific and intraspecific genetic diversity of free‐living ciliates (Lynn, 2008). In the last 10 years, several other regions of the small (SSU rDNA) and large ribosomal DNA (LSU rDNA) subunits have been tested for their suitability as DNA barcodes for ciliates (Santoferrara & McManus, 2020). Furthermore, with the invention of high‐throughput sequencing (HTS) technologies, hypervariable regions of the SSU rDNA have become popular as molecular markers for ciliates and other microbes (Gimmler et al., 2016; Stoeck et al., 2014).

Collectively, this suite of novel molecular technologies has revealed a surprising genetic diversity of planktonic ciliates in marine and freshwaters, but the meaning of this diversity remains unclear (Caron & Hu, 2018). For instance, the Tara Oceans dataset yielded ciliate diversity based on OTUs (operational taxonomic units) that surpassed the known number of morphospecies by six‐fold (de Vargas et al., 2015). However, there remains a continued need to ground‐truth OTUs against the more traditional morphology‐based methods (see Clamp & Lynn, 2017; Warren et al., 2017). For instance, Santoferrara et al. (2020) indicated that what is inferred as a species using morphology may not be inferred using OTUs. Furthermore, “omics” techniques are currently only semi‐quantitative; i.e. they do not provide accurate estimates of the organismal abundance or biomass (d'Alelio et al., 2019). Issues associated with using metabarcoding data to infer ciliate abundance have been addressed by Mahé et al. (2017) and Fu and Gong (2017). In summary, we now know that many more ciliate genotypes “are out there,” but the crucial questions for ecological forecasting are “do they represent species” (Caron & Hu, 2018), “how many are there?”, and “what are they doing?” (Bittner et al., 2010).

### Parameterizing rates (in situ and in vitro experiments)

In conjunction with determining ciliate abundance, biomass, and diversity (as outlined above), rate measurements were needed. What and how much do ciliates eat? How fast do ciliates reproduce? What predators eat ciliates? Which key biotic and abiotic factors affect these rates? The resulting combination of rates and standing stocks could then provide understanding and allow forecasting (see Synthesis and application, below).

Determining ecological rate measurements is, however, always fraught with the balance between accuracy and precision. In situ experiments—often difficult to conduct on planktonic systems—accurately represent natural conditions but tend to lack precision, while laboratory‐based in vitro experiments on culturable taxa provide precise measurements, but their reflection of in situ process remains uncertain. Likewise, in situ measurements tend to provide discrete values under conditions defined by the local environment, while laboratory experiments offer flexibility in estimating rate dependencies. Starting in the 1980s and continuing to this day, the study of planktonic ciliates has successfully coupled these two approaches providing a means to rigorously evaluate and characterize rates. Not surprisingly, both approaches developed following substantive methodological breakthroughs (Figure 1).

For in situ experiments, a number of methods were developed—mostly in the 1980s and often adapted from phytoplankton and zooplankton methods—to maintain captured samples under natural conditions (Table 1). These methods, many of which are still used, included suspended containers with porous membranes, allowing ciliates and their prey to be incubated in near‐natural conditions and bottles filled with recently collected water that are gently rotated (e.g. on‐board a ship) to prevent sedimentation (Båmstedt et al., 2000; McManus, 1993; Runge & Roff, 2000). Within these incubations, ciliate and prey populations change over time, and rate measurements can be determined. Numerous variations on this theme have been developed, but the most notable one of these is the “dilution technique” (Landry & Hassett, 1982), which although having some possible drawbacks (Agis et al., 2007) has, for 40 years, been widely used to determine ciliate grazing rates in situ (Båmstedt et al., 2000; Calbet & Saiz, 2013; Landry, 1994).

Although many ciliates are easy to grow and systematic culturing of freshwater ciliates began over 100 years ago (Pinheiro & Bols, 2013), planktonic species—from marine and freshwaters—initially proved difficult to culture, and remain challenging, likely due to clonal decline of cultures (Montagnes et al., 1996). In the 1980s, a breakthrough was the development of methods to culture planktonic ciliates (Gifford, 1985, 1993; Soldo & Brickson, 1993). These studies at least, in part, answered the first of our questions: many planktonic ciliates consume and grow on a range of easily cultured nanophytoplankton. This led to a host of experiments in the following decades that addressed the remaining questions, typically through determining functional and numerical responses (i.e. grazing and growth rates vs. prey abundance, respectively; Weisse, 2017; Weisse et al., 2016) and how they vary under a range of biotic and abiotic conditions, in particular assessing thermal performance (Montagnes et al., 2008). Numerous innovative approaches have been developed to conduct these experiments; far more than we have space for.

### Parameterizing rates through indices

Indices have been explored to predict the rates of ciliates from static in situ measurements, including DNA content (Wickham & Lynn, 1990) and the frequency of dividing nuclei in a population (Coats & Heinbokel, 1982), but by far the most common—and most pragmatic—of these has been cell size, typically scaled to temperature. The arguments underlying these predictive functions are that larger ciliates grow slower (although they may have higher grazing rates), and increased ambient temperature increases rates (Hansen et al., 1997; Montagnes, 1996). Coupled with size‐based estimates of biomass (Menden‐Deuer & Lessard, 2000) and sized‐fractionated field estimates of abundance, these estimates of growth rate can provide predictions of in situ ciliate production; i.e. the elaboration of biomass over a defined time, in a defined area or volume (Montagnes et al., 2010).

New advances are identifying functional genes and linking measurement of their activity, to organismal activity (Carradec et al., 2018; d'Alelio et al., 2019). Measuring these genes, or their activity may then act as an index of organismal function. For instance, meiosis‐specific or meiosis‐related genes are well known from ciliates (Chi et al., 2013; Dunthorn et al., 2017). For macroorganisms, sexual recombination can enhance genetic diversity and allelic combinations among different loci, facilitating adaptation to environmental change (Becks & Agrawal, 2012). In the late 17th century, King and van Leeuwenhoek had already observed sex (conjugation) in ciliates (Bell, 1988; Dini & Nyberg, 1993), and sex is recognized as essential for many ciliates and specifically planktonic ciliates (Bell, 1988; Montagnes et al., 1996). However, the frequency of sex remains virtually unknown in planktonic ciliates (Weisse, 2006, 2008). An extensive empirical study and theoretical considerations suggest that the frequency (or index) of conjugation is < 1% in marine ciliates (Lucchesi & Santangelo, 2004; Weisse, 2014). High rates of conjugation (> 25%) were inferred indirectly for a pond‐dwelling population of *Tetrahymena thermophila* (Doerder et al., 1995) and directly observed in an oligo‐mesotrophic lake (Weisse & Sonntag, 2016). Clearly, this issue is of relevance for adaptive responses of ciliates to climate change, and indices to assess it would be beneficial.

Importantly, in contrast to traditional methods, many of the novel genetic tools do not require cultured material or laborious microscopical observation. However, identification of gene function requires reference genomes that are, in the case of ciliates, only available for some well‐studied species, such as *Tetrahymena* and *Paramecium* (Johri et al., 2017; Stover et al., 2006); genomes and transcriptomes from these species may only poorly represent the diversity of the pelagic, often rare, ciliates (del Campo et al., 2014; Keeling et al., 2014). Relative to other protists such as phytoplankton dinoflagellates, there has been a notable lag in metatranscriptomics studies on ciliates. Before we can apply “omics” methodologies to planktonic ciliates in more detail, a much better understanding of the newly discovered genes is needed for a deeper appreciation of their functional diversity and actions (Caron et al., 2017).

## SYNTHESIS AND APPLICATION

We began this examination of ciliates by suggesting that the objective of most plankton ecology is forecasting i.e. making predictions. In this final section, we very briefly indicate how our current knowledge and understanding of planktonic ciliates have been applied to this end. We then focus on the single most pressing ecological issue of our time, climate change, and suggest the next steps—conceptual and methodological—that we see as now being required of planktonic ciliate ecologists.

### Plankton models

It would be presumptuous to even try to outline the extent of plankton models here (see Carlotti et al., 2000; Ghyoot et al., 2017). We recognize that over the last 100 years, planktonic ciliates have been incorporated into models ranging from conceptual graphics (e.g. Figure 2), to simple trophic interactions (Montagnes et al., 2008), to complex numeric simulations (Heneghan et al., 2020). Model complexity and structure will depend on the question being addressed, but the modeler's adage of “rubbish in, rubbish out” will always apply. The last four to five decades have provided substantial data that have improved our estimates of rate parameters and constrained the ranges of state variables, but there remains considerable scope for increasing the accuracy of these. Above, by indicating where we have been, we also offer—or at least allude to—some directions for what should be done next. In the following section, we provide more directed guidance.

### Current trends and future directions to address climate change

Over the last 150 years, there has been a number of conceptual and technological developments that have encouraged researchers and funders to pursue the study of ciliates (Figure 1, Table 1). Our brief review illustrates that better estimates of abundance, diversity, and biomass coupled with rate measurements have led to the incorporation of ciliates in ecosystem models, providing recognition of their importance. Despite the well‐established roles of ciliates in planktonic systems, our greatest concern is the current, rather retroactive, lack of appreciation by many plankton ecologists and ecosystem modelers that ciliates are neither heterotrophic phytoplankton nor tiny zooplankton (see Initial awareness and conceptualization). Compared to the enlightenment of the 1980s to 2000s (Figure 1), ciliates, currently, often seem to be relegated to be junior zooplankton, rather than central figures in the microbial food web (Figure 2). With this concern in mind, and in terms of understanding the impacts of climate change, we see the following as priorities (listed in no particular order) in the technological and conceptual advancement of planktonic ciliate research:

1. Our current understanding is heavily biased by studies on temperate ciliates, from inshore waters and some selected lakes. Climatic hot spots, such as the Arctic and Antarctic oceans, remain understudied.
2. Ciliates cannot be collected (spatially or temporally) the way mesozooplankton or phytoplankton are. Ciliates are temporally and spatially patchy, and bloom dynamics—which will be exacerbated by shifting thermal landscapes—need to be properly assessed to appreciate the impacts of climate change on food web dynamics and carbon flux. Simply adding ciliate measurements onto a suit of existing zooplankton/phytoplankton‐based sampling is far from appropriate. Instead, improving methodologies such as molecular identification of single cells and imaging flow cytometry to recognize taxa would be a giant step toward improving our understanding.
3. New, culture‐independent genetic methods should cast more light on the functional ecology of the rare biosphere (Caron & Countway, 2009; Dunthorn et al., 2014; Weisse, 2014). Currently, though, it remains unknown if the large number of rare ciliates is important for the stability and functional resilience of microbial communities or is mostly functionally redundant at the community level.
4. Ciliates have generation times on the order of days and exhibit high phenotypic and genetic diversity. Consequently, genetic adaptation (i.e. microevolution) and shifts in population composition are likely to occur rapidly in response to shifts in climate; much more rapidly than for mesozooplankton. Conjugation in ciliates, as with other organisms, is often stimulated by adverse conditions. Whether climate change increases the frequency of conjugation and facilitates adaptive processes in ciliates is an open question. Concerning functional genes in general, it is crucial that the reference database is improved to reliably link gene sequences (OTUs) to known ciliate species.
5. Marine and freshwater ciliates share a relatively long common evolutionary history, with the transition to freshwater being relatively recent (Forster et al., 2012). Therefore, it seems plausible to assume that evolutionary and ecophysiological differences between marine and freshwater ciliates are minor, but the potential for their rapid adaptation to changing environmental conditions (see 4, above) suggests otherwise. Surprisingly, there has been little attempt to appreciate or understand the ecological implications of the potential differences. Importantly, it should be established if marine and freshwater ciliates display different reaction norms to environmental drivers mediated by climate change.
6. Unlike mesozooplankton, due to mixotrophy, ciliates cannot be placed into the “heterotroph” box (Stoecker et al., 2017). Not only will they potentially change their trophic status, but temperature shifts may alter the ratio at which auto‐/heterotrophic processes occur (Yang et al., 2016).
7. Laboratory experiments and field studies are needed that target specific climate‐change‐related questions (e.g. acclimation vs. adaptation of the temperature tolerance of ciliate species) and the response of the ciliate community to extreme events. Furthermore, these approaches should develop and apply automated measurements of abundance and biomass with the molecular assessment of functional gene expression.
8. Even within the ciliate assemblage, there is considerable trophic diversity, leading to as yet understudied interactions (e.g. predation/cannibalism, parasitism, chemical communication, and cooperation). These interactions may be altered by thermal mismatches and should be investigated experimentally with ecologically relevant species.
9. Based on the rapidly accumulating empirical evidence allowing better parameterization, models marrying genomics, gene expression (transcriptomics), and (trait‐based) function, coupled with climate change scenarios, are needed to predict how pelagic ciliates will adapt to global warming and ocean acidification, and how this adaptation will impact carbon flux in the pelagic realm.

In short, research focused on planktonic ciliates requires continued academic attention and financial support to develop the new technologies that are arising and to marry those methods and their future findings with existing techniques and data. Only by doing so will we continue to develop new conceptual understandings of how ciliates will respond to climate change, ultimately providing tools for forecasting.
